# Editorial: Understanding the impact of loneliness and social isolation on the health of older adults with cancer

**DOI:** 10.3389/fragi.2026.1930051

**Published:** 2026-07-22

**Authors:** Keith M. Bellizzi, Rebecca M. Saracino

**Affiliations:** 1 Department of Human Development and Family Sciences, University of Connecticut, Storrs, CT, United States; 2 Memorial Sloan Kettering Cancer Center, New York, NY, United States

**Keywords:** health, loneliness, older adults with cancer, social connection, social isolation

## Introduction

As the population of older adults living with and beyond cancer continues to expand, increasing attention has focused on the social and psychosocial factors that shape survivorship, functional aging, and quality of life. Among these factors, loneliness and social isolation have emerged as critical, yet often under-examined, determinants of health. Although loneliness and social isolation are related constructs, they represent distinct dimensions of social experience, one reflecting perceived social disconnection and the other objective limitations in social contact or support. Both have been linked to poorer mental and physical health outcomes, diminished quality of life, functional decline, and mortality in older populations. In the context of cancer and aging, these challenges may be amplified by symptom burden, treatment effects, caregiving demands, changing social roles, and disruptions to identity and daily life.

This Research Topic, *Understanding the Impact of Loneliness and Social Isolation on the Health of Older Adults with Cancer*, brings together diverse contributions that collectively advance understanding of how social disconnection influences survivorship experiences, psychosocial wellbeing, cognitive health, and intervention development among older adults with cancer and their care partners. Importantly, the articles included in this collection move the field beyond simply documenting prevalence toward identifying mechanisms, measurement approaches, modifiable targets, and innovative intervention strategies.

### Psychosocial consequences of social disconnection

Several articles in this Research Topic underscore the profound psychosocial consequences associated with loneliness and social isolation. Using data from the Study of Women’s Health Across the Nation (SWAN), Avis et al. examined associations between loneliness, social isolation, and health outcomes among older cancer survivors and women without a cancer history. Their findings demonstrate that loneliness and social isolation were strongly associated with poorer mental health-related quality of life and greater depressive symptoms, regardless of cancer survivorship status. These findings reinforce the broader public health importance of social disconnection while highlighting that cancer survivors may experience vulnerabilities similar to those observed in the general aging population.

Extending this work, Kitrel et al. explored psychosocial correlates of social isolation among distressed older adults with cancer receiving psychotherapy. Their analyses demonstrated that social isolation was independently associated with depressive symptoms even after accounting for anxiety, demoralization, and demographic characteristics. The study further highlights the complex emotional landscape surrounding social disconnection in cancer survivorship and identifies potentially modifiable psychosocial targets for future intervention efforts.

The importance of social relationships within the context of close partnerships is highlighted by Winters-Stone et al., who examined loneliness among couples coping with breast and prostate cancer. Their findings reveal that loneliness is highly prevalent not only among survivors, but also among care partners, and is associated with anxiety, depressive symptoms, pain, and impaired social functioning. Particularly notable is their exploration of dyadic exercise interventions, which suggests that shared physical activity and group-based participation may help reduce loneliness and strengthen social connection among couples navigating cancer together.

Complementing these findings, Arrato et al. examined social wellbeing among older breast cancer survivors receiving adjuvant endocrine therapy. Their study identified self-efficacy for symptom management as a strong correlate of social wellbeing, even after accounting for pain-related factors. These findings suggest that confidence in managing treatment-related symptoms may help preserve social engagement and reduce vulnerability to loneliness during long-term survivorship.

### Intervention development and opportunities for prevention

Beyond documenting psychosocial associations, this Research Topic also emphasizes innovation in intervention development and prevention strategies. Dugan et al. used a participatory design approach to develop a peer-support intervention for employed breast cancer survivors experiencing social disconnection. Survivors identified changes in self-concept, symptom-related withdrawal, and unsupportive social responses as key drivers of disconnection. The resulting multi-component intervention, which incorporates peer-led support, digital resources, and opportunities for social engagement highlights the value of survivor-centered approaches that directly integrate lived experience into intervention design.

Leach et al. further broaden the intervention landscape through a perspective article proposing group-based exercise as a potentially synergistic strategy to reduce loneliness and social isolation while simultaneously supporting cognitive health among older cancer survivors. Their conceptual model integrates exercise-related neurobiological benefits with the social engagement fostered through group participation, offering a compelling framework for future translational research targeting both cognitive decline and social wellbeing.

### Advancing measurement and methodology

Measurement and methodological advancement also emerge as central themes across this collection. Marziliano et al. describe the development of the Social Isolation Risk Scale (SIRS), a novel screening instrument designed specifically for head and neck cancer survivors. By focusing on cancer-specific drivers of social isolation, the SIRS represents an important step toward improving identification of patients at heightened risk for social disconnection in busy clinical settings.

At a broader methodological level, Merluzzi et al. provide a timely review of psychometric network analysis as an emerging framework for studying loneliness, social isolation, and social support in oncology populations. Their review illustrates how network analytic approaches may help disentangle complex relationships among symptoms, coping resources, and psychosocial functioning while identifying potential intervention targets that are not easily captured using traditional latent variable models.

### Implications for survivorship and healthy aging

Collectively, the articles in this Research Topic highlight several important themes. First, loneliness and social isolation are multidimensional experiences that intersect with emotional health, physical functioning, cognition, and survivorship quality of life. Second, these experiences extend beyond patients themselves and may profoundly affect intimate partners and caregivers. Third, social disconnection is not merely a correlate of poor outcomes, but a potentially modifiable target for intervention. Finally, advancing this field will require stronger measurement approaches, longitudinal research designs, and innovative interventions that integrate psychosocial, behavioral, and social dimensions of survivorship care.

As the population of older cancer survivors continues to grow, addressing loneliness and social isolation will become increasingly important for promoting healthy aging, preserving quality of life, and improving survivorship outcomes. As survivorship care increasingly emphasizes healthy aging and functional wellbeing, social connection should be recognized not as ancillary to cancer care, but as a core dimension of comprehensive survivorship and geriatric oncology practice.

